# The rocky road to prosocial behavior at work: The role of positivity and organizational socialization in preventing interpersonal strain

**DOI:** 10.1371/journal.pone.0193508

**Published:** 2018-03-01

**Authors:** Stefano Livi, Annalisa Theodorou, Marika Rullo, Luigi Cinque, Guido Alessandri

**Affiliations:** 1 Department of Social and Developmental Psychology, University of Rome “Sapienza”, Rome, Italy; 2 Institut de Psychologie, University of Lausanne, Lausanne, Switzerland; 3 Department of Psychology, University of Rome “Sapienza”, Rome, Italy; Monash University, AUSTRALIA

## Abstract

Among relevant consequences of organizational socialization, a key factor is the promotion of organizational citizenship behaviors toward individuals (i.e. OCBI). However, the relation between organizational socialization and OCBI has received little attention. This study tests the validity of a moderated mediation model in which we examine the mediating effect of a decreased interpersonal strain on the relationship between organizational socialization and OCBI, and the moderation role of a positive personal resource in reducing interpersonal strain when an unsuccessful socialization subsists. A cross-sectional study was conducted on 765 new recruits of the Guardia di Finanza–a military Police Force reporting to the Italian Minister of Economy. Findings confirm our hypothesis that interpersonal strain mediates the relationship between organizational socialization and OCBI. The index of moderated mediation results significant, showing that this effect exists at different levels of positivity. Theoretical and practical implications for promoting pro-organizational behaviors are discussed.

## Introduction

Organizational citizenship behaviors (OCB) refer to “individual behaviors that are discretionary, not directly or explicitly recognized by the formal reward system, and that in the aggregate promote the effective functioning of the organization” ([1], p.1). Among the different kinds of OCB, OCBI are defined as those devoted to sustain, encourage, empathizing, and helping co-workers [2]. Whereas the benefits of these behaviors for the recipient are quite clear, empirical studies have reported their positive effects for the performer of OCBI [3] and for the larger organization itself [4]. Some scholars argued that considering themselves as organizational insiders foster the probability to implement OCBI [5–7]. Presumably, this occurs because an insider is more likely to assume the responsibility of the duties carried by organizational citizenship, and as a result, to spontaneously participate in the group’s life, and behave in a cooperative way (see [5], p. 315 and [6], p. 880). In sum, there is reason to hypothesize a role for organizational socialization, the process by which an individual acquires values, skills, expected behaviors, and social knowledge necessary to assume an organizational role [8,9], in the prediction of the frequency with which different workers perform OCBI in the workplace. However, besides a few exceptions [10,11], the relation between OCBI and levels of organizational socialization has not received the attention it deserves.

**Fig 1:**
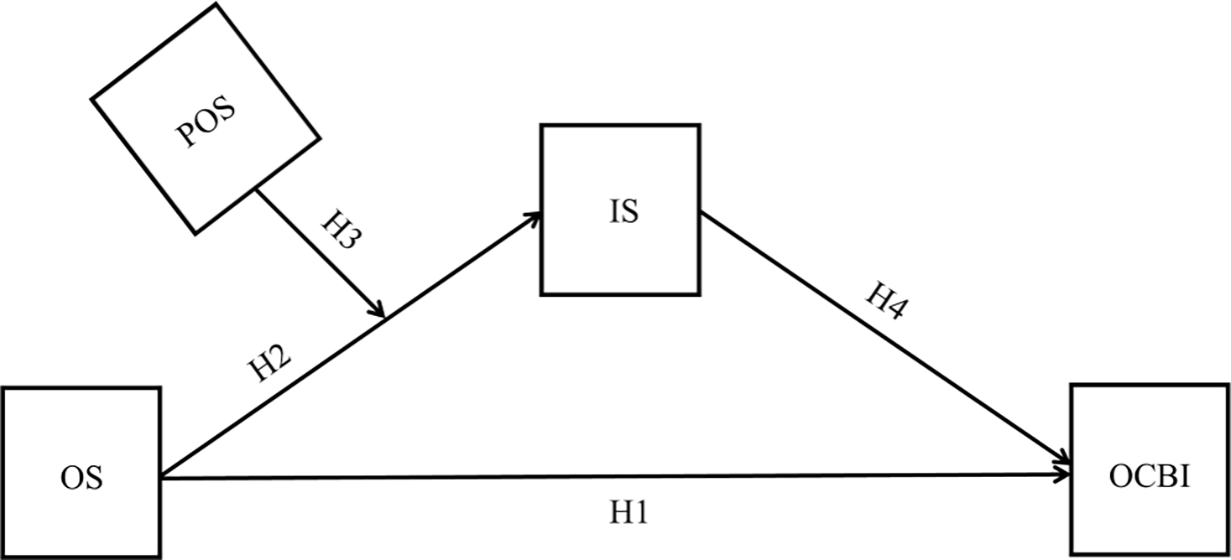
The hypothesized model. Legend: OS: Organizational socialization; POS: Positivity; IS: Interpersonal strain; OCBI; Organizational citizenship behaviors toward individuals.

The purpose of this study is to explore the predictive validity of a theoretical moderated mediation model (see Fig 1) in which the individual level of organizational socialization predicts OCBI, through the mediation of a reduced interpersonal strain [12], defined as: “a specific disengagement reaction towards demanding interpersonal interactions and social pressures, through which the person creates emotional and cognitive distance from other people at work” ([12], p. 878). The basic idea is that organizational socialization helps individuals to manage the organizational interpersonal context. Accordingly, the lower the level of organizational socialization, the higher the relational burden perceived by newcomers. Since newcomers are active participants in their adjustment process to the new work environment [13,14], this model also acknowledges a privileged role to positivity, a personality trait that refers to a positive outlook towards oneself, life, and future [15–17], conceptualized as a personal resource that is able to compensate for low organizational socialization levels and act as a protective factor against the development of interpersonal strain. Below, we delineate the model describing each of the five theoretical relationships hypothesized in more detail.

As mentioned before, the first relationship hypothesized is the one between organizational socialization and OCBI. While OCB are naturally discretionary behaviors, they are nonetheless linked to expectations and norms more or less explicitly associated with different organizational roles [18,19], and to the implicit rules and forecasts that inform interpersonal interactions [5,20]. As a consequence, to engage in OCB people need a general knowledge of the formal and informal expectations associated with their own duties and obligations towards the organization and the other persons at work. Thus, it is not surprising that performing OCBI carries a cost to the individuals. These costs are likely to be particularly high for newcomers that are not familiar with the new organizational environment, including their formal and informal role and social expectations [21,22].

Newcomers are newly hired employees who have recently joined the organization [23]. As such, through the organizational socialization process, new workers are called to undertake a role transition [24]: from being organizational outsiders to become insiders [25]. Organizational socialization represents indeed the process by which this role transition occurs. Evidences support that a successful organizational socialization process reduces organizational costs, by preventing early turnover [26], and by enhancing workers’ satisfaction, commitment, and performance [27]. Although under some conditions newcomers may engage in citizenship behaviors [28], generally when individuals adapt to the organization, they develop indeed a sense of satisfaction and commitment towards it, two indicators that are also important predictors of OCBI [29]. According to the studies mentioned so far, newcomers would not possess the feeling of membership with the organization, the complete awareness connected to their in-role and extra-role behaviors, and the job satisfaction and commitment necessary to engage in OCBI. Hence, we suggest that individuals with lower levels of organizational socialization are less likely to perform OCBI (see Fig 1, Hypothesis 1).

Our second theoretical argument is that organizational socialization can play a role in the prediction of interpersonal strain. Entering for the first time in an organization exposes newcomers to several unexpected stressors. Often, no extra time is given to newcomers to progressively adapt to the new environment, and newcomers are called from the beginning to respect their obligations and duties, while not proper knowing yet what others are expecting from them [30]. As a consequence, the immersion in this new and foreign context can be disorienting and demanding, and the newcomer may experience what is known as a “*reality shock”* [22]. Thus, it is natural that in the early stages of the organizational socialization process the different *socialization agents* (i.e., co-workers, supervisors, subordinates, clients, and customers) play a central role [31]. Different socialization agents can help the new hires in several ways: for example in (1) interpreting events experienced in the workplace [8,13]; (2) guiding and supporting newcomers in learning their role [8,13]; (3) providing access to information and resources [32–34]; and (4) offering social support [23,35]. Most importantly, colleagues and supervisors represent important sources to learn informal expectations about desired OCBI [20,36].

More in general, there is evidence that networking with co-workers and gaining acceptance by insiders can facilitate the adjustment to the organization [37]. Empirical studies show that feelings of being accepted are positively related to important organizational outcomes such as higher commitment and job satisfaction [38], higher performance [39], and negatively related to turnover [39]. Unfortunately, developing relationships with co-workers and being included in a social group represents a critical task for newcomers [40]. In sum, when observed by the individual perspective, the process of organizational socialization entails a kind of work *demand* that requires a “…sustained physical and/or psychological effort” and it is “…associated with certain physiological and/or psychological costs” ([41], p. 501). The individual level of organizational socialization, instead, represents a *resource*, since it proves to be “…functional in achieving work goals, reduce job demands and the associated physiological and psychological costs or stimulate personal growth, and development” ([41], p. 501). In our study, we hypothesize that high levels of organizational socialization can prevent interpersonal strain (see Fig 1, Hypothesis 2).

Our third relationship hypothesized takes into account that, despite the obvious link between levels of socialization and perceived stress, some people are more inclined than others to develop stress-related symptomatology [42]. Individual characteristics appear, in this sense, to act as personal resources, exerting a protective role on individuals’ health. The Conservation of Resources Theory [43], for example, individuated in the trait of positivity a key individual resource to cope with stressful situations (see [43], p. 519). Accordingly, positivity helps to minimize the psychological consequences associated with the experience of stress. The author states that a positive stable evaluation of the self and the world makes people perceive future as more predictable. This sense of control makes them feel confident that they will master future hard circumstances (e.g. losses or failures).

Likely, the more recent and systematic account of positivity has been offered by Caprara and colleagues [15–17]. According to the authors, positivity represents a positive view of oneself, life and future that predisposes individuals to confront with challenges and difficulties of life [16]. Positivity is a stable, trait-like, evaluative disposition that represents a common latent factor determined by three constructs: self-esteem [44], optimism [45], and life satisfaction [46]. By leading individuals to see events as predictable and generally occurring in one’s best interest [43], positive workers are better equipped to cope with stress and difficulties, and thus have greater personal resources to invest in their work. Therefore, positivity leads individuals to perceive their working conditions as more favorable, their work goals as more reachable, and to reduce the impact of the challenges and stressors resulting from daily experiences at work. These beneficial effects of positivity are also important resources within work groups [47].

In our study, we expected that the higher the level of personal resources (i.e., positivity) the lower the observed relationship between organizational socialization and interpersonal strain. This occurs because individual positivity may act as a personal resource and alleviate the burden ingenerated on the individual by a still imperfect organizational socialization. Positive individuals are indeed expected to perceive potentially noxious stimuli less negatively, and to possess a higher confidence to be able to counteract negative emotions associated with social distress [15,48]. By making them more secure in their future capacity to deal with different kind of social situations, the most difficult too, it is possible that positivity can foster the individuals’ tendency to be open to new social experiences in the workplace, without fearing them, and to be more willing and friendly with others at work, even with complete strangers. In the event of social pressures, positivity can protect newcomers from taking the distances. Based on these considerations, the third hypothesis of the study provides for the dispositional trait of positivity to moderate the relation between individuals’ level of organizational socialization and interpersonal strain (see Fig 1, Hypothesis 3).

In our model, the fourth relationship hypothesized is the one between interpersonal strain and OCBI. Empirical studies attest to a significant and negative link between perceived stress (and associated negative affects) and OCBI [49]. Moreover, it is well known that individuals are more likely to perform OCBI when they experience positive emotions [50]. Furthermore, as hypothesized by Borgogni et al.[12], the recurrent experience of negative emotions at work may lead workers to the development of feelings of interpersonal strain, and to the nurturing of a sense of detachment by colleagues. Individuals who withdraw from social interactions are more likely to perform less OCBI because they progressively came to consider interpersonal relationships as distressful, harming and, in a word, dangerous. These considerations led us to hypothesize a direct negative link between interpersonal strain and OCBI (see Fig 1, Hypothesis 4).

All in all, assuming that positivity moderates the association between organizational socialization and interpersonal strain implies that positivity beliefs conditionally influence the strength of the indirect relation between organizational socialization and OCBI. More concretely, positivity is hypothesized to act as the mechanism able to lower or enhance the likelihood of organizational socialization to enact OCBI (see Hypothesis 5). This hypothesis, in turn, makes our hypothetical model a "moderated mediation model" [51], in which the effect of an independent variable (organizational socialization) on the outcome (OCBI), and the partial effect of the mediator (interpersonal strain) on the outcome (OCBI), depends on the levels of another variable (in the present case, positivity). In summary, study’s hypotheses are presented below (see also Fig 1):

H1: *Organizational socialization is positively associated with the frequency of individuals’ OCBI in the workplace*.
H2: *Higher organizational socialization levels predict lower interpersonal strain at work*.
H3: *Positivity moderates the relation between organizational socialization and interpersonal strain*.
H4: *Interpersonal strain negatively predicts a higher frequency of OCBI at work*.
H5: *Positivity moderates the indirect effect of organizational socialization on OCBI (through interpersonal strain)*.

## Method

### Participants and procedure

Data used in this study came from a cohort of individuals that applied and were selected for attending the first year of one of the military academies belonging to Guardia di Finanza, a military Police Force reporting directly to the Italian Minister of Economy and Finance. All participants filled out the questionnaire because they were involved in a larger plan implemented by the military force, aimed at supporting the adjustment of new recruits into the military context. Therefore, the sample represents a complete cohort of military enrolled in the first year of the academy (i.e., response rate = 100%), and it is naturally representative of the population of military attending the Guardia di Finanza academies in Italy in the year 2016.

Participants were 765 new recruits of the Guardia di Finanza interviewed through a web-based survey after two months of their first entrance into the military schools, of which 620 were males (81% of the sample) and 145 (19%) were females. The average age was 25.05 (SD = 3.24). Not all individuals were at their first military experience, in fact, they had 3.15 years average of previous military experiences (from 0 to 10). Only 278 (36.3%) recruits have not had a previous involvement in the military contest, by contrast, 356 (46.5%) had a preceding experience in the army, 34 (4.4%) in the navy, 29 (3.8%) in the military aviation, 27 (3.5%) in the Guardia di Finanza, 26 (3.4%) had two military experiences in two different armed forces, and 15 (2%) in the port authority. Regarding recruits’ level of education, 91.5% of the respondents had a high school diploma, 5.4% had a bachelor’s degree, 2.6% had a master’s degree, and 0.5% had a junior high school diploma. Data on organizational socialization, OCBI, interpersonal strain, and positivity were collected by a web-based survey. Data collection took place at the military schools’ computer labs situated in five different military academies across Italy. We interviewed 100 recruits at a time. Participants were verbally informed about research scopes, questionnaire content, and voluntary participation so that they could give their informed consent for participating. They were assured anonymity and protection of sensible data, emphasizing that information use was intended for research purposes only. The method applied in the study was approved by the Ethical Committee of the Department of Social and Developmental Psychology of the University of Rome “Sapienza”. Data were anonymized prior to analysis in order to protect participants’ privacy and anonymity and are available at http://dx.doi.org/10.17605/OSF.IO/XQE4S.

### Measures

#### Organizational socialization

To estimate newcomers’ levels of organizational socialization we used the Organizational Socialization Questionnaire (OSQ, see S1 File): this instrument is composed by 18 items measuring levels of the construct based on three socialization dimensions [52]. The first one, named *identification*, is measured by 6 items (e.g. “*My organization is an important part of me*”). The second factor, named *competence*, is measured by 6 items (e.g. “*I’m still learning all of the work tasks of my job*”). The third factor is named *acceptance by co-workers* and is measured by 6 items (e.g. “*I feel completely accepted by my colleagues*”). The questionnaire already shown high convergent validity (r = .77; p < .001) with other socialization questionnaires, such as the CAS (Content Area of Socialization) [53,54]. Respondents answered all items by using a 5-point Likert scale, from 1 (*strongly disagree*) to 5 (*strongly agree*), and reverse items (*n* = 12) were recoded. Following previous studies, we used in all analyses the total scale score. Higher individuals’ scores on this factor were considered evidence of higher levels of organizational socialization. The Cronbach’s alpha was .81

#### Interpersonal strain

Interpersonal strain was assessed by the 6-item scale by Borgogni et al. ([12], see S1 File). Item examples are “*At work*, *I treat others in a cold and detached manner*”, “*Sometimes when I’m working*, *it happens to me to mistreat someone*” and “*At work*, *I feel irritated by other people*”, and responses were ranging from 0 (*never*) to 6 (*daily*). High scores on the scale indicate high emotional and mental distance from others at work. Cronbach’s alpha for this scale was .85.

#### Positivity

Positivity was evaluated by the Positivity Scale by Caprara and colleagues ([55], see S1 File). The instrument consists of 8 items (e.g., “*I am satisfied with my life*”, “*I generally feel confident in myself*”, “*Others are generally here for me when I need them*”), one of which is reversed (i.e. “*At times*, *the future seems unclear to me*”) and has been recoded. Responses ranged from 1 (*strongly disagree*) to 5 (*strongly agree*), and high scores show high levels of positivity. The Cronbach’s alpha was .84.

#### OCBI (organizational citizenship behaviors toward individuals)

Newcomers’ OCBI was measured by the corresponding dimension of the Williams and Anderson’s scale([2], see S1 File), composed of 7 items (e.g. “*Helps others who have been absent*”, “*Takes time to listen to co-workers’ problems and worries*”, and “*Passes along information to co-workers*”). Responses ranged from 1 (*strongly disagree*) to 5 (*strongly agree*), and high scores correspond to a high frequency of OCBI. The alpha coefficient was .72.

### Data analysis

To test our hypotheses, we used a moderated mediation regression model, using Hayes’ PROCESS macro for SPSS ([56], model 1 and 7). The model was composed of two successive steps, as introduced by Baron and Kenny [57]. In the first step positivity, socialization, and their interaction were posed as predictors of interpersonal strain, while in the second step we regressed OCBI on interpersonal strain, controlling for the effect of organizational socialization. In addition, a model was tested in which we introduced in both steps age, sex, educational qualification, and years of previous military experience as covariates, but relations of the main variables remained significant.

To verify the statistical significance of the model, we used the index of moderated mediation: this index tests if two paths, consisting of the indirect effect calculated at each of the two levels of the moderator (i.e. one standard deviation above and below the mean), are statistically different [58]. As recommended by MacKinnon, Lockwood, and Williams [59], 95% confidence intervals were calculated using the bias-corrected bootstrap (based on 5000 bootstrap samples): confidence intervals that not include zero indicate that the indirect effect is significantly different from zero at p < .05. In order to facilitate the interpretation of the moderation analysis, positivity and interpersonal strain variables were mean-centered before being included in the model.

## Results

Table 1 shows the correlations among organizational socialization, interpersonal strain, positivity, and OCBI, as well as means and standard deviations for all the variables. As expected, organizational socialization showed strong and negative correlation with interpersonal strain, a strong and positive correlation with positivity, and a moderately high positive correlation with OCBI. In turn, interpersonal strain showed a moderately high negative correlation with positivity and OCBI. Finally, positivity showed moderately high positive correlation with OCBI. All the correlation coefficients resulted statistically significant (see Table 1).

**Table 1:** Correlations, means and standard deviations for measured variables (N = 765). In diagonal alpha coefficients.

	1	2	3	4
**1. Organizational socialization**	(.81)			
**2. Interpersonal strain**	−.49**	(.85)		
**3. Positivity**	.51**	−.44**	(.84)	
**4. OCBI**	.29**	−.29**	.35**	(.72)
**Mean**	3.88	.29	4.67	3.98
**Standard deviation**	.47	.54	.41	.55

Note

** p < .01

*Legend*: OCBI: Organizational citizenship behaviors towards individuals.

### Moderated mediation analysis

As explained above, for testing our moderated mediation model, we ran two separate regression models. The first model regressed interpersonal strain (the outcome variable) on socialization, positivity, and the interaction between socialization and positivity. Results from this model are presented in the first column of Table 2, and attested: (1) a significant and negative prediction of interpersonal strain by organizational socialization (*β* = −0.37, *SE* = 0.04, *p <* 0.001 [95% CI −0.44, −0.30]), confirming hypothesis 2; (2) a significant and negative prediction of interpersonal strain by positivity, although the effect size in this case is not as strong as the previous relation (*β* = −0.13, *SE* = 0.04, *p* < 0.001 [95% CI −0.21, −0.05]); and (3), most importantly, a significant interaction between organizational socialization and positivity (*β* = 0.14, *SE* = 0.03, *p <* 0.001 [95% CI 0.09, 0.19]), confirming hypothesis 3.

**Table 2:** Effects of the predictors on interpersonal strain and OCBI as criteria.

	Criteria					
	Interpersonal strain			OCBI		
***Predictors***	***β***	**SE**	**95% CI**	***β***	**SE**	**95% CI**
**Constant**	−.07*	.03	[−0.14, −0.01]	.00	.03	[−0.07, 0.07]
**Organizational socialization**	−.37**	.04	[−0.44, −0.30]	.19**	.04	[0.11, 0.27]
**Interpersonal strain**	—	—	—	−.20**	.04	[−0.28, −0.12]
**Positivity**	−.13**	.04	[−0.21, −0.05]	—	—	—
Organizational socialization*Positivity	.14**	.03	[0.09, 0.19]	—	—	—
**F**	115.55**			47.6**		
**R**^**2**^	.31**			.11**		

Note

** p < .01

* p < .05.

**Fig 2:**
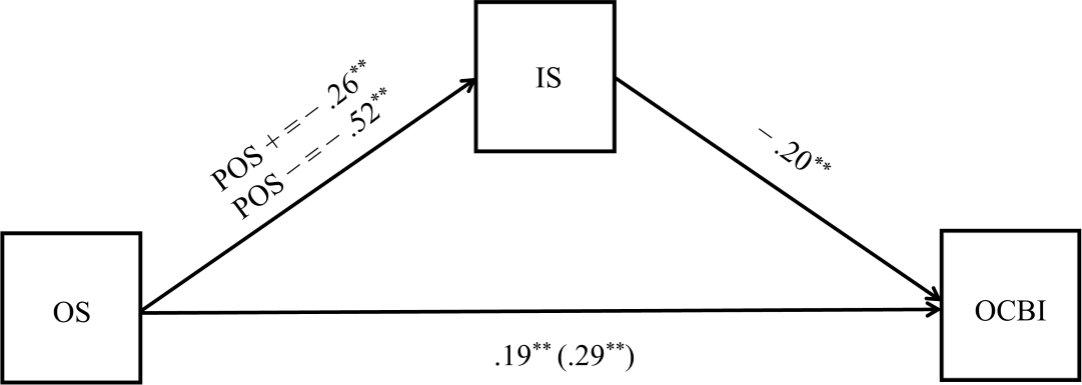
Results of the moderated mediation model. Note: ** p < .01 Legend: OS: Organizational socialization; POS: Positivity; IS: Interpersonal strain; OCBI; Organizational citizenship behaviors toward individuals.

**Fig 3:**
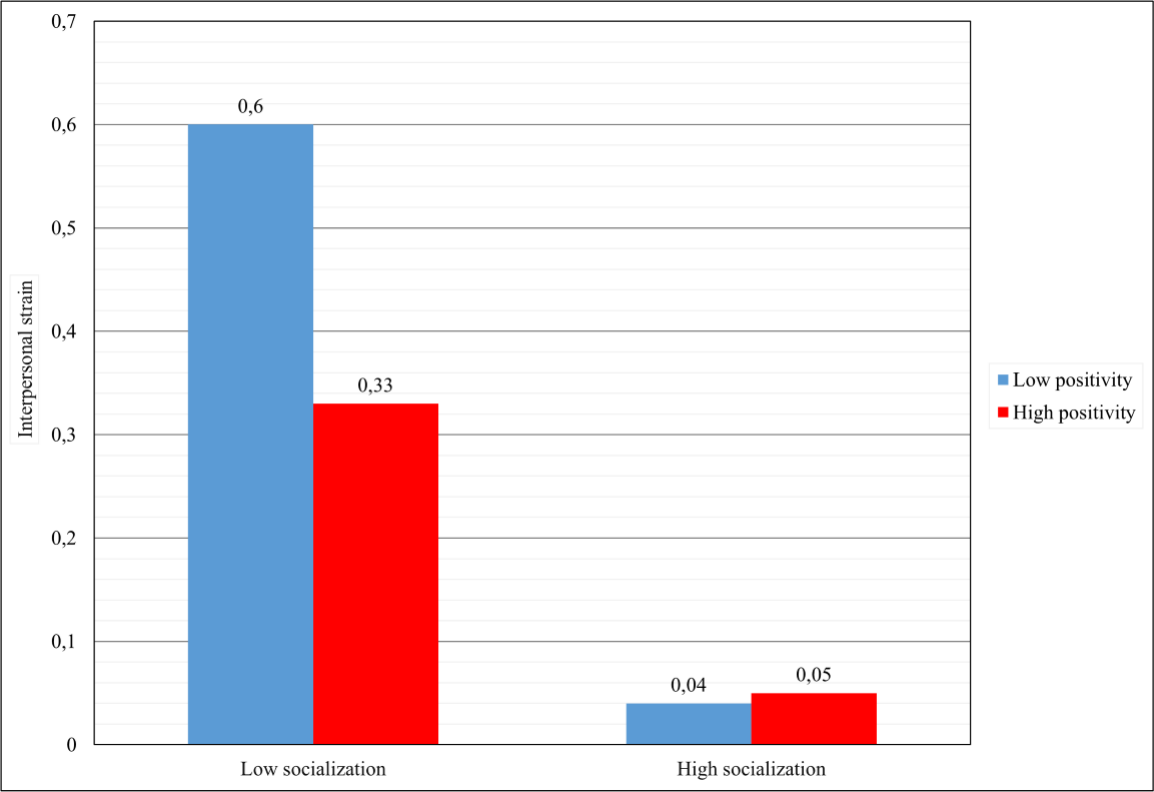
Simple slope analysis of the interaction between positivity and organizational socialization on interpersonal strain.

To better understand the nature of this interaction, simple slope analysis was conducted. Results revealed that the negative relation between socialization and interpersonal strain was stronger when the individuals’ positivity was low (*β* = −0.52, *SE* = 0.05, *p <* 0.001 [95% CI −0.60, −0.43]) than when individuals’ positivity was high (*β* = −0.26, *SE* = 0.04, *p <* 0.001 [95% CI −0.34, −0.18]). These results are presented graphically in Fig 2 and fully confirmed hypothesis 3. Indeed, when recruits are highly socialized, the interpersonal strain is low, no matter how the intensity of their positivity is. By contrast, when socialization is low, positivity makes the difference, reducing considerably the interpersonal strain only when is high (see Fig 3). The model accounts for a significant proportion of variance, namely 31% of the outcome (see Table 2).

The second regression model specified OCBI as the outcome variable, and (1) organizational socialization and (2) interpersonal strain as independent variables. Results from this model are presented in the last two columns of Table 2. Interpersonal strain significantly predicted OCBI, confirming hypothesis 4, and there was a significant prediction of OCBI from organizational socialization. To investigate the hypothesis that the indirect effect of organizational socialization on OCBI through interpersonal strain changes at different levels of positivity (hypothesis 5), we computed the significance of this indirect effect for high (i.e. one standard deviation above mean) and low (i.e. one standard deviation below mean) positivity. Results from this analysis confirmed our hypothesis that the indirect effect between socialization and OCBI, thought interpersonal strain, is strong and significant when positivity is low (*β* = 0.10, *SE(Boot)* = 0.04, [95% BOOT-CI 0.04, 0.17]), and it is weaker, although still significant, when positivity is high (*β* = 0.05, *SE(Boot)* = 0.02, [95% BOOT-CI 0.02, 0.09]). Fig 2 shows these effects graphically. Finally, providing further support to our model, the index of moderated mediation resulted significant (*index* = −0.03, *SE(Boot)* = 0.01, [95% BOOT-CI −0.06, −0.01]). Again, the model accounted for a significant proportion of variance, explaining 11% of the outcome (see Table 2).

## Discussion

Socializing in a new organizational environment carries a cost to the individual in terms of personal resources investment. Although less acknowledged, workers’ socialization carries a cost to the organization too, paid in terms of a reduction of person's voluntary commitment with all the organizational tasks or informal duties that are not part of the contractual tasks. In this paper, we investigated the reduction of the voluntary availability with colleagues and the larger social environment that workers experience as a consequence of increased feelings of interpersonal strain, that arises from low levels of socialization. Our results, based on a large sample of 765 new recruits, assign a crucial role to positivity as the buffer able to reduce, although not to break, this maladaptive circle.

While we investigated the mechanisms linking socialization to OCBI in a sample of newcomers, organizational socialization did not occur only in the initial stage of a worker career. Indeed, in face of nowadays labor market instability, employees are often forced by the organizations to modify their work and social environment in order to keep up with the incessant changing circumstances. Under these conditions, for their part organizations are called upon to confront with the effects of these changes on human resources and their influence on individual behaviors [60]. The nature of OCBI is such that they result malleable to modification, and thus they appear susceptible to change in response to specifically implemented interventions [29]. Thus, knowing the mechanisms linking workers’ socialization to OCBI, conditional on feelings of interpersonal strain and positivity, offers a potential strategy of intervention useful in all situations in which an organization passes through a phase of major changes, or a worker transfers from an organization to another.

Moving to discuss more in detail the results of our study, according to the first hypothesis, we observed a positive relationship between individual levels of organizational socialization and frequency of OCBI. As anticipated by our theoretical reasoning, being socialized, namely being identified with the organization, feeling competent to engage in one’s own role tasks, and perceiving acceptance from other co-workers, enhance the likelihood to implement prosocial behaviors at work [9,52]. Similarly, newcomers could exhibit lower effective OCBI because they are not feeling yet part of the organization, they do not know the formal and informal social expectation of the new work environment, and do not possess proper levels of job satisfaction and commitment, necessary to engage in these behaviors. Although previous studies have repeatedly suggested such a possibility [10,11], our is the first, as we know, to offer a direct test of the proposition that organizational socialization directly operationalized as a function of identification, competence, and acceptance, is a predictor of organizational citizenship behaviors towards individuals as defined by Williams and Anderson [2].

Low levels of socialization naturally seem to increase the feelings of discomfort and disengagement in the relationships with people at work, probably reflecting a need of the worker to withdraw from a challenging, and in part unknown, interpersonal environment. This result accords with other findings showing that organizational socialization acts as a positive resource in preventing burnout in the workplace [14,61]. Our theoretical model further acknowledges that individual resources, in terms of organizational socialization, do not operate in isolation, but tend to often behave in combined synergies [15,62]. This idea was represented in the model by the interaction between levels of socialization and positivity.

Results offered support to this idea. Indeed, having a positive view of one’s self, life and future proved to buffer the onerous effects of interpersonal strain in the event of low socialization. This result fits with the large literature highlighting the importance of positive personality traits offering to the individual a stable feeling of control on the reality, of hope in the future and of satisfaction with what an individual has done in the past, in sustaining the individual’s coping with hard times [17,43,63]. However, an attentive reading of our results suggests the primacy of organizational socialization over positivity. While positivity may modulate the (direct) relation between levels of socialization with stress and (the indirect) with OCBI, in the presence of high levels of socialization findings show that there is no difference in how high positive and low positive individuals experience interpersonal strain. Thus, while it is true that in the picture returned by the data, resources originated by the individual and by the group compensate each other, these latter seem to perform a key function. It is important to note that all the relations remained unvaried after controlling for several covariates previously shown to play a major role in the military setting.

### Limitations

The study presents several limitations that deserve consideration. First, the cross-sectional research design threats the internal validity of the study. In order to explore the causality of the observed relations, in future studies, the longitudinal or the experimental design are needed. Second, data were collected on a specific armed force, the Guardia di Finanza, and future studies could test the hypothesized model in others armed forces allowing a generalization of the results to the extended military context. Third, the particularity of the sample limits the generalizability of the results to other contexts apart from the military environment. More studies could investigate the reliability of the model in older samples, namely in a sample of workers experiencing different processes of organizational socialization (e.g. role transition, moving from an organizational part to another, moving and start another job) and in other organizational contexts, to corroborating the generalizability of the model. For example, future studies can consider interviewing participants in different timings of the recruits’ adjustment to the academia, in order to test the temporal stability of the model. Lastly, another limitation is constituted by the data collection methods. First, the choice of group testing might have had an influence on fraudulent responses, since individuality is denied [64]. Second, web-based surveys could have been problematic [65]. In our case, instruments validity can depend on the interaction between participants and the computer. For instance, the degree of computer skills could play a role in the understanding of instructions, survey characteristics, as the number of question per page, could have a role in increasing tiredness of participants, and the background color could influence responses to emotionally charged statements. For these reasons, future studies need to use different data collection methods.

### Practical implications

Acknowledging the full implications of our results is important in order to plan interventions aimed to support newcomers during earlier phases of organizational socialization. Our model, indeed, assigns a value to both organizational socialization and positivity in the prediction of OCBI via interpersonal strain. However, the indications offered by our model for each construct are different. While the results obtained for positivity seem to suggest that organizational socialization might be easier for positive individuals, they underline that organizational socialization carries a cost (although minor) also for them. Thus, effective interventions should aim not only to the activation of positive individual resources (see for an example, an intervention aimed to develop individual resources) but also to improve the fit of the individual with his/her environment by promoting socialization (for instance, improving socialization tactics). To be sure, mixed interventions (i.e., interventions aimed to activate resources and to improve socialization) seem doomed to produce the better results. In addition, military academies and organizations could evaluate and control in more times the individual level of socialization at first stages of adjustment and, accordingly, improve socialization tactics.

### Conclusions

In conclusion, this study suggests that newcomer’s adjustment to the organization indirectly promotes organizational effectiveness in terms of OCBI. Future research needs to consider other mediators and moderators of the observed relations, in order to enlarge the knowledge on the phenomenon. Furthermore, findings open to the salience of socialization with the group for the development of stress-related symptoms. Possible future avenues to better understanding this result, may be testing the above model using a group perspective and thus collecting multilevel data on teamwork variables. For instance, in order to be accepted, newcomers in the early stages of socialization tend to conform to the group norms [66]: group norms (i.e. cooperative or competitive) have a central role in enhancing (or decreasing) the frequency of OCBI [67]. Thereby, we believe that this kind of design may allow consideration of group and context variables (such as group norms) that was not included in the present study.

In conclusion, despite some limitations, we believe that our theoretical model, strongly corroborated by our data, has much to offer to the understating of the mechanism that links organizational socialization to OCBI via the stress process and in combination with positive personality traits.

## Supporting information

S1 File Appendix. Measures used in the study.
(PDF)
