# Supplementary material for: The rocky road to prosocial behavior at work: The role of positivity and organizational socialization in preventing interpersonal strain
Source: PLoS One. 2018 Mar 1;13(3):e0193508. doi: 10.1371/journal.pone.0193508 (PMC5832254; doi:10.1371/journal.pone.0193508)
Supplement: S1 File — Measures used in the study. (PDF) [file pone.0193508.s001.pdf]

Appendix

Measures used in the study.

Organizational Socialization Questionnaire

1. I have learned how to carry out my work-related activities and duties well.

2. I know perfectly well how I should behave with my colleagues.

3. For some time, I no longer identify myself with the values of my organization (reverse scored).

4. In the past, I felt closer to my colleagues (reverse scored).

5. I’m still getting to know my colleagues (reverse scored).

6. I’m gradually starting to identify with my organization (reverse scored).

7. I’m considered “one of the group”.

8. I identify completely with my organization.

9. When changes are introduced in the way work-related activities are carried out, it is hard for me to learn the new procedures (reverse scored).

10. I often have the impression of being left on the sidelines by my workgroup (reverse scored).

11. I’m doing my best in order to one day feel completely identified with my organization (reverse scored).

12. I’m still learning all of the work tasks of my job (reverse scored).

13. I feel completely accepted by my colleagues.

14. My organization is an important part of me.

15. I don’t always agree with the values of my organization (reverse scored).

16. In the past, I attempted to keep myself more informed on the skills required to do my job efficiently (reverse scored).

17. My colleagues still don’t know me well (reverse scored).

18. I still don’t feel skilled enough in the tasks I have to perform in my organization (reverse scored).

Interpersonal strain (Borgogni et al., 2015)

1. At work, I feel more comfortable keeping distance from others.

2. At work, I find myself to be insensitive to other people's problems.

3. At work, I treat others in a cold and detached manner.

4. At work, I'm not particularly interested in what happens to others.

5. Sometimes when I'm working, it happens to me to mistreat someone.

6. At work, I feel irritated by other people.

Positivity (Caprara et al., 2012)

1. I have great faith in the future.

2. I am satisfied with my life.

3. Others are generally here for me when I need them.

4. I look forward to the future with hope and enthusiasm.

5. On the whole, I am satisfied with myself.

6. At times, the future seems unclear to me (reverse scored).

7. I feel I have many things to be proud of.

8. I generally feel confident in myself.

OCBI (Williams and Anderson, 1991)

1. Helps others who have been absent.

2. Helps others who have heavy work loads.

3. Assists supervisor with his/her work when not asked.

4. Takes time to listen to co-workers’ problems and worries.

5. Goes out of way to help new employees.

6. Takes a personal interest in other employees.

7. Passes along information to co-workers.
